# Social Class Identity, Public Service Satisfaction, and Happiness of Residents: The Mediating Role of Social Trust

**DOI:** 10.3389/fpsyg.2021.659657

**Published:** 2021-06-01

**Authors:** Xiaogang Zhou, Shuilin Chen, Lu Chen, Liqing Li

**Affiliations:** ^1^School of Economics and Management, East China Jiaotong University, Nanchang, China; ^2^School of Economics and Management, Jiangxi Science and Technology Normal University, Nanchang, China

**Keywords:** social class identity, social trust, public service satisfaction, happiness of residents, structural equation model

## Abstract

Happiness is the eternal pursuit of mankind and is also the ultimate goal of social governance and national development. Based on data from the Chinese General Social Survey, this study used a structural equation model to analyze the influence of social class identity and public service satisfaction on the happiness of residents. The effect of public service satisfaction and social trust between social class identity and residents’ happiness was tested using the Monte Carlo method. The empirical results show that social class identity, social trust, and public service satisfaction all had a significant positive impact on residents’ happiness. The influence coefficients of social class identity on the happiness of residents and on the satisfaction degree of public service for those born in the1970s group were greater than the 1980s and 1990s groups. The influence coefficients of social class identity on the happiness and public service satisfaction of non-agricultural household residents were greater than those of agricultural household residents. Therefore, to improve the happiness of residents, we should make new breakthroughs in the equality of quality and the quality of public services, promote the integration of urban and rural areas, highlight key areas of rural development, and increase the construction of basic public services for agricultural household residents.

## Introduction

The notion that “it is hard to get a good son in a poor family” has become a hot topic in all sectors of society in recent years and highlights the imbalance between the horizontal opportunity structure and the vertical intergenerational structure of the social class and public education (Khor and Pencavel, 2010; Di, 2014; Li and Zhu, 2015). The fundamental difference between different social classes lies in the degree of occupation or control of social resources. In different social systems and social cultures, the value of social resources is different; therefore, social classes are also different. In a society with a stable social strata, the influence of social resources (politics, economy, culture) on people’s social status is relatively certain. Therefore, the more the social resources at disposal, the higher the current social class. The higher the social class, the higher the ability to stimulate individual enthusiasm and creativity, which is conducive to promoting the harmonious development of society and the effective improvement of residents’ happiness (Dai and Yi, 2012).

However, with large-scale and high-speed class differentiation, reorganization, and changes in social class relations in society, changes in the reference group made up of people that habitually associate and identify with behavior can easily affect people’s relative deprivation, which, in turn, affects residents’ happiness. Some local governments have begun to consciously regard the improvement of residents’ happiness as an important governance task. Many scholars have explored the factors influencing residents’ happiness from different perspectives. For example, a positive correlation has been found between income levels and subjective personal happiness, and the happiness of people on a low-income is lower than that of people on a high-income (Clark et al., 2008; Lu and Wang, 2011; Pu and Yuan, 2019; Ye and Zhang, 2020). Socio-demographic characteristics, such as residents’ health, marital, and employment status, have an impact on residents’ happiness (Blanchflower and Oswald, 2004; Dolan et al., 2008). Some scholars have also combined income and air pollution data to study how residents’ happiness changes with environmental conditions (Welsch, 2006; Brereton et al., 2008; Sun and Lu, 2019).

Unlike previous studies on the relationship between social class identity and residents’ happiness, which have mostly used the urban and rural dual social structure theory, the current study is based on the reference group theory, social capital theory, and the happiness economics theory with the assumption that public service satisfaction and social trust are mediating variables in the relationship between social class identity and residents’ happiness. Based on the Chinese Comprehensive Social Survey (CGSS) data, this study empirically examines the influence of social class identity, public service satisfaction, and residents’ happiness, and explores the causal mediating effect between social trust and public service satisfaction.

The gains made in the current study, based on previous research, may lie in the following. First, this study constructs a reasonable and self-consistent overall logical analysis framework based on the reference group theory and happiness economics, and incorporates social class identity, social trust, public service satisfaction, and residents’ happiness into a unified discussion to compensate for the deficiencies of separate discussions in the extant literature. Second, this study refines the different birth years into the 1960s, 1970s, 1980s, and the 1990s groups and the different types of households into agricultural and non-agricultural to identify the different effects of social class identity, social trust, and public service satisfaction on residents’ happiness in different birth years and different types of households. Third, structural equation modeling is used to conduct an in-depth study of the 1960s, 1970s, 1980s, and 1990s groups and agricultural and non-agricultural household groups. The difference between social class identity and residents’ happiness is more practical, taking into account the interaction between multiple variables.

## Hypothesis Development and Research Model

### Effect of Social Class Identity on Residents’ Happiness

It is generally believed that people at the top of both the economy and society are happier than those at the bottom because they have more social resources and can meet more of their needs. Social class identity refers to an individual’s perception of their position in a social class structure (Jackman and Jackman, 1973). It reflects an individual’s integration in social interactions and focuses on the individual’s psychological acceptance, adaptation, and internalization of the social environment and clusters. According to the reference group theory, in the process of comparing one’s self to others, satisfaction is determined based on the comparison between one’s own situation and the situation of other members of the group (Funatsu, 2009). Social class identification focuses on the interpersonal interaction and relationship formation patterns formed by individuals in a social environment, and positive social class identification helps individuals improve their happiness, cognitive security, and social trust. Therefore, people with a higher social class identity can experience more positive emotions, relatively speaking, while people with a lower social class identity are more likely to experience more negative emotions. Ultimately, due to the differences caused by social class identity, emotional experiences will affect people’s happiness. People often compare their current situation with their previous situation, or with that of others. If they find that they receive less or relatively less resources than they deserve, a psychological gap will occur. Once people think that they are relatively disadvantaged, even if their own situation has been greatly improved on an absolute level, their social class identity in the group will be relatively reduced, resulting in the feeling of “relative deprivation,” and their sense of happiness will decrease accordingly (Stouffer et al., 1949).

Whether an analysis is based on a cross-country sample (Helliwell et al., 2015; Mizobuchi, 2017) or a specific country or region (Könönen et al., 2007; Barger et al., 2009), social class identity and its positive flow has a significant positive impact on the happiness of residents. Moreover, the happiness effect of changes in the social class of rural residents is more sensitive to that of urban residents (Run, 2012; Hu et al., 2019), while children are more sensitive to the decline in their parents” professional status (Lu and Zhang, 2014; Li, 2017). Changes in social class identity and expectation has a significant effect on happiness (Fischer et al., 2009; Zhang and Yang, 2017; Wang M., 2019). When the socio-economic status and the cautious identification of urban and rural residents remain equal, there is no significant difference in the happiness between the residents (Liang and Wang, 2014; Xu and Chen, 2017; Yin et al., 2019). Burton (2008) conducted a study on Canadian adolescents aged 12–15 and found that economic resources, relative socio-economic status, and social relations affected their happiness. On one hand, family income has an important relationship with the happiness distribution of young people at the bottom of society, but it is not associated with the happiness of those at the top of society. On the other hand, relative socio-economic status and social relations with peers is related to the distribution of teenagers’ happiness at the top of society but not with those at the bottom. Accordingly, we propose the following hypothesis:

**Hypothesis 1:** Social class identity positively affects residents’ happiness.

### Effect of Social Class Identity on Public Service Satisfaction

From the perspective of micro-society, different social classes have different cognitions and needs for public services because of the inequality of the social division of labor and resource allocation. Therefore, public service satisfaction also differs. Social class is rooted in both the context of the material substance of social life and in people’s subjective perception of their class identity. This is a core aspect of how people view themselves and connect with the social world (Kraus et al., 2012). Lin’s (2005) theory of social resources states that the higher a person’s social class, the greater their opportunities to obtain social resources. The level of social class determines the quantity and quality of social resources owned by individuals. Satisfaction with public services is essentially a manifestation of personal cognition or social attitudes. The level of social class identification is often a subjective reflection of the level of possession of various objective resources, including public services, and the amount of satisfaction with public services will directly affect the differences in public service satisfaction. The combined effect of objective material resources and subjective perception has led to the formation of different social classes. People in the same social class form a relatively stable cognitive tendency due to shared experiences (Zani, 2012). Different stratum groups, especially high- and low-stratum groups, have certain differences in their cognition of society, which is reflected in the relationship between class identity and public service satisfaction. People with a low-level identity are more likely to be affected by their surrounding environments. When public service resources are unevenly distributed among different classes, people with a low-level identity tend to have fewer public service resources and lower public service quality than those with a high-level identity (Robinson-Neal, 2009). Therefore, they attribute their disadvantage and their lower-class status to external conditions such as insufficient public services and unbalanced resource supply. Compared to those who identify with the higher class, the lower-class more easily make negative evaluations of public service satisfaction. People with a lower social class identity have a lower sense of access to public services and have lower satisfaction. People with a higher social class identity have a stronger sense of acquisition and have higher satisfaction.

Many factors influence the satisfaction of public services. From the perspective of the macro-social structure, different social classes show different levels of satisfaction with public services provided by the government (Xu, 2018; Cohen et al., 2020; Liu and Qu, 2020). In the extant research, the definition of social class has two main research perspectives: objective socio-economic status and subjective class identification (Xu and Yuan, 2019; Lou and Lu, 2020; Vazquez and Cubbin, 2020). From the perspective of objective socio-economic status, the main indicators used by academic circles are income, education, and occupation. In terms of income indicators, existing studies have shown that high-income groups are generally more satisfied with public services than low-income groups (Zhang et al., 2011; Sun, 2016). However, no absoluteness has been observed. It is not simply the case of the higher the income, the higher the satisfaction with public services; it is also the relative deprivation from a comparative perspective (Ma and Liu, 2010; Yuan and Xia, 2012; Liu et al., 2019). From the perspective of educational indicators, in a study on the political attitudes of the middle class in China, Zhang (2008) found that groups with higher levels of education had lower levels of trust in the local government, and satisfaction with the work of the local government was also low. Simultaneously, he pointed out that the process of modernization and the popularization of education have strengthened the awareness of those that are educated regarding social reflection enhancing their pursuit of rights beyond material life. From the perspective of occupational indicators, one’s occupational status is usually determined by acquired factors, such as education and work skills. Li and Li (2007) compared migrant workers with urban workers and found that although migrant workers are lower than urban workers in occupational class, their satisfaction with the government was generally higher than that of urban workers, indicating a more positive social attitude. In short, people with a higher professional status are less satisfied with public services. Past research has shown that people’s objective class status is not consistent with their subjective class identification, and this inconsistency is also reflected in the evaluation of public service satisfaction (Assila et al., 2016; Chen and Fan, 2019). In the research on the impact of subjective class identification on public service satisfaction, the higher the subjective class identification, the higher one’s satisfaction with public services (Zhang, 2005). Accordingly, we propose the following hypothesis:

**Hypothesis 2:** Social class identity positively affects public service satisfaction.

### Effect of Social Class Identity on Social Trust

Different social classes agree that individual’s amounts of disposable resources differ, which leads to different abilities to resist risks. The more resources an individual has, the stronger their ability to resist risks, and the better their ability to face the risks brought about by interacting with strangers. Therefore, individuals with a higher objective economic status can bear the possible loss of trusting strangers (Brehm and Rahn, 1997; Tan, 2016). In short, the higher an individual’s social class identity, the higher the level of social trust. Some studies have supported the above hypothesis; for example, Delhey and Newton (2005) measured trust by questionnaires and found that an individual’s class identity, number of resources, wealth possessed, and level of trust were significantly positively correlated. Other studies have used large sample data survey methods and found that an individual’s objective social class was positively correlated with general trust (Gheorghiu et al., 2009; Hamamura, 2012; Ao et al., 2013; Gao, 2015). Giddens (1991) believed that the better the internal and external conditions of an individual, the more optimistic they are. The more positive and comfortable they are about life, the more they rely on themselves instead of being restricted by resources, and the more willing they are to trust others. Conversely, people that have negative internal and external conditions do not trust others easily. Wang and Liu (2002) also have the same view; that is, the proportion of possible losses among all resources owned by an individual will directly affect their trust level. Luhmann (2005) put forward the “disaster threshold” concept, which states that this possible loss may be devastating for the individual and will affect their level of social trust. Everyone has their own bottom line of risk. If the loss caused by the untrustworthiness of another cannot be borne by themselves, the individual will not choose to trust others. The level of this bottom line is related to the actual number of resources available to an individual. The fewer resources a person has, the lower their disaster line and risk tolerance, and the less willing they are to take risks and trust others. Accordingly, we propose the following hypothesis:

**Hypothesis 3:** Social class identity positively affects social trust.

### Effect of Public Service Satisfaction on Social Trust

Satisfaction with public services refers to a subjective evaluation of whether public services meet people’s expectations or their daily needs (Wang Y., 2019). Li and Hao’s (2008) survey of four places in the Guanzhong region found that villagers’ satisfaction with the township government services included six aspects: land allocation and expropriation, social security, education, medical insurance, infrastructure construction, and pension security. These aspects positively affected the trust of the township government. Hu et al. (2011) found that citizens’ satisfaction with government affairs and urban governance services had a positive impact on government trust. Based on a survey of Shanxi Province’s townships, Che (2012) concluded that citizens’ satisfaction with compulsory education, public health, and public security had a positive impact on the trust of local township governments. He and Tang (2012) analyzed the data of 300 prefecture-level cities’ e-government assessments and found that public services can indirectly affect government trust by affecting government transparency. Therefore, it is feasible to improve public services and increase government transparency through e-government, thereby increasing government trust. Yi’s (2013) survey also found that the general public’s satisfaction with public services and products provided by local governments had a positive impact on enhancing social trust. Peng and Li (2018) found that people’s satisfaction with local government’s public services (e.g., primary and secondary education, elderly care, employment, medical care, housing, etc.) all had a positive impact on social trust. If people are satisfied with the public services provided by the government, they will think that their expectations have been fulfilled. According to social exchange theory, there is an exchange relationship between people and the government. The starting point of the exchange is the people’s expectations of the government’s capabilities and behaviors. People provide the government with resources, trust, votes, and so on in exchange for the government’s quality public services. Therefore, the more people are satisfied with the public services provided by the government, the higher their trust in the government, and the people who trust the government will return the government with higher responsiveness and tolerance, which is conducive to the healthy development of society. Accordingly, we propose the following hypothesis:

**Hypothesis 4:** Public service satisfaction positively affects social trust.

### The Mediating Effect of Social Trust

Trust is a psychological state and refers to an individual’s willingness to accept the corresponding risks based on the positive expectations of others’ intentions and behaviors (Rousseau et al., 1998). Social trust refers to the trust of individuals in the general sense of the existence of other objects in society. This dimension of trust is often used when discussing its significance in social development (Jivraj and Nazroo, 2014). The construction of social trust networks is a proposition that has attracted much attention. Under the influence of factors such as individualization, utilitarianizing, and marketisation, the phenomenon of trust destruction between people is very common, and even the phenomenon of “killing familiarity” occurs. Individuals and groups can fall into the so-called “crisis of confidence.” Social trust requires the participation of power, market, society, and other entities to ensure the supply of capital, environment, and factors for its existence to jointly create a social order supported by formal systems and informal resources. A study found that trust is related to many factors and is affected by institutional culture, public resource supply, economic development status, and so on (Shin and Jo, 2015). Simultaneously, it has a significant impact on factors such as the relationship between the government and the public, the level of social collaborative governance, and the level of people’s physical or mental health (Liu, 2013). Some scholars believe that social trust is closely related to residents’ happiness (Rodríguez-Pose and Von Berlepsch, 2014), and others have used World Value Survey (WVS) China data to demonstrate the positive correlation between social trust and residents’ happiness (Yuan and Xia, 2012), which subsequent studies have confirmed. Ma (2018) used a multi-layer linear model to explore the impact of social trust on residents’ happiness and found that social trust, membership, and other social capital could significantly improve residents’ happiness. Chen et al. (2019) found that social trust had a significant positive impact on residents’ happiness, and the higher the level of social trust, the stronger the happiness of the residents. Accordingly, we propose the following hypothesis:

**Hypothesis 5:** Social trust positively affects residents’ happiness.

**Hypothesis 6:** Social trust has a mediating effect between social class identification and residents’ happiness.

### The Mediating Effect of Public Service Satisfaction

The function of public services is to build a safe, stable, and fair social environment. These services not only improve the protection of residents’ properties and personal safety but also ensure social fairness and justice, which can improve residents’ happiness (Zhao et al., 2019). Public services are non-exclusive and fair, and perfect services can enable everyone to enjoy the services of individual countries. Regardless of class and wealth, lifestyle improvements can be obtained fairly through public services, which is conducive to narrowing the social gap, reducing residents’ sense of social injustice, increasing their sense of gain, and improving their sense of happiness. The increase in government spending on education and public medical care can also help increase residents’ happiness, as imperfect infrastructure and unequal opportunities have a happiness deprivation effect across regions, especially between urban and rural areas. Moreover, social instability and environmental pollution also have different effects on different income groups. Low-income groups lack the ability to reverse compensation and thus experience a significant decline in happiness, which directly leads to the unfairness of the environmental welfare in the same area (Sun and Mei, 2019; Dong et al., 2020). Thus, people choose to live in cities so that they can enjoy better public services. If the development of the public service industry is difficult to coordinate with the accelerating urbanization process, the ensuing “urban disease” will lead to a decline in residents’ happiness (Xia and Lu, 2015).

Happiness economics believes that the government’s provision of cheap public goods will increase the relative income of residents, and thus the residents’ sense of happiness will also increase (Li, 2019). Complete public education, medical and health care, elderly care, and convenient public transportation can reduce people’s living costs so that the middle- and lower-classes can still enjoy a higher quality of life, even with relatively low incomes. When people can meet or improve their quality of life and standards that they cannot achieve through public services, their happiness will inevitably be improved. Public governance issues such as unemployment, income gaps, living environment, and social security also have a significant impact on residents’ subjective happiness (Oswald, 1997; Tao, 2019). There have been many domestic and foreign studies involving empirical research on public services and residents’ happiness, but they are often measured by dimensions such as the adequacy and equilibrium of public service resources (Dolan et al., 2013; Ferreira and Busatto, 2013; Huang, 2013; Case and Deaton, 2015; Wang Y., 2019). There is a lack of studies that consider public education, medical and health care, housing security, and other aspects of public services as intermediate variables. Zhou et al. (2015) believes that the quality of public services has a significant impact on residents’ happiness, that residents attach great importance to public service quality and service methods, and that public service satisfaction is a standard for measuring public service quality. Therefore, public service satisfaction has a significant impact on residents’ happiness, and the higher the public service satisfaction, the higher the residents’ happiness. Ding (2016) used effective micro-survey data of 3,949 residents in 28 provinces in China and found that public service factors, such as basic housing security and medical and health care, had a significant positive impact on residents’ happiness. Wu (2017) conducted a survey of residents in 56 cities in China and found that the supply of basic public services directly affected residents’ subjective happiness. Hou (2018) explored the impact of public service satisfaction on subjective happiness and found that public service satisfaction and subjective happiness had a significant positive impact. Therefore, there is still room for discussion on the impact of public service satisfaction on residents’ happiness. Accordingly, we propose the following hypothesis:

**Hypothesis 7:** Public service satisfaction positively affects residents’ happiness.

**Hypothesis 8:** Public service satisfaction has a mediating effect between social class identification and residents’ happiness.

**FIGURE 1:**
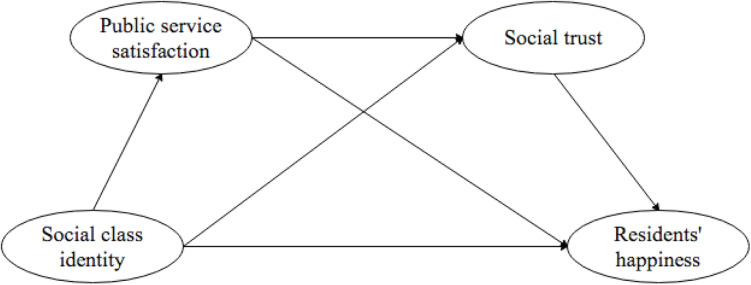
Research model.

Our theoretical research model is depicted in Figure 1.

## Method

### Participants and Procedure

The data in this study were from a national large-scale multi-level stratified sampling survey project jointly led by RENMIN University of China and the Hong Kong University of Science and Technology, referred to as the Chinese General Social Survey (CGSS) 2015. The database is currently recognized authoritative data for China scholars studying residents’ happiness, economic behavior and other issues. The project started in 2003 and is China’s earliest national, comprehensive, and continuous academic survey project. It has been conducted 9 times so far. A total of 125 counties or districts, 478 villages, 500 streets, townships or towns, about 1,000 residents’ committees, and more than 40,000 families in village committees were selected from 28 provinces. Through a household survey, 10,968 valid questionnaires were completed. According to the variables examined in this study, samples that contained “refusal to answer,” “do not know,” and “not applicable” were eliminated. Finally, a random valid sample of 6,352, distributed across 28 provinces, was obtained (Tibet and Xinjiang were not included). Participants’ demographic data were as follows: 3,113 (49.0%) were men and 3,239 (51.0%) were women. In terms of age, 857 (13.5%) were aged under 30 years, 2,095 (33.0%) were aged 31–49 years, 2,420 (38.1%) were aged 50–69 years, and 980 (15.4%) were over 70 years. In terms of household status, 4,102 (64.6%) were agricultural households and 2,250 (35.4%) were non-agricultural households. In terms of marital status, 777 (12.2%) were unmarried and 5,575 (87.8%) were married. In terms of academic qualifications, 2,226 (35.0%) were below the primary school level, 1,916 (30.2%) were the junior middle school level, 1,176 (18.5%) were secondary or high school level, and 1,034 (16.3%) were above the university level.

### Measures

#### Residents’ Happiness

According to positive psychology and social comparison theory, subjective well-being includes not only the perception of happiness and quality of life, but also the comparison of economic and social status with others. From this, the following variables were selected. In the residents’ happiness selection questionnaire, the items for “In general, do you think your life is happy?” were “very/complete/very unhappy” (= 1), “relatively unhappy” (= 2), “not happy or unhappy/between happiness and unhappiness/average” (= 2), “relatively happy” (= 4), and “very/total happiness” (= 5). The items for “What do you think of your current physical health?” were “very unhealthy” (= 1), “relatively unhealthy” (= 2), “fair” (= 3), “relatively healthy” (= 4), and “very healthy” (= 5). The items for “Which class does your family’s economic status belong to?” were “much lower than average” (= 1), “lower than average” (= 2), “average” (= 3), “higher than the average level” (= 4), and “much higher than the average level” (= 5).

#### Social Class Identity

According to social identity theory, social identity mainly comes from group membership or qualification, and is rooted in people’s subjective perception of their own class identity. From this, the following variables were selected. In the social class identification latent variable selection questionnaire, the following four items were the observed variables: “Which level do you think you are currently at?”; “Which level do you think you were at 10 years ago?”; “Which level do you think you will be in 10 years?”; and “Which level do you think your family was at when you were 14 years old?.” To ensure the consistency of the variable values, this study conducted conversion processing on levels 1–10 of the CGSS 2015: [9–10] = 5, [7–8] = 4, [5–6] = 3, [3–4] = 2, and [1–2] = 1.

#### Public Service Satisfaction

Public services include public education, medical and health care, housing security, social security and so on. From this, the following variables were selected. The latent variables of public service satisfaction were selected from the questions on “public education satisfaction,” “medical and health satisfaction,” “housing security satisfaction,” “social management satisfaction,” “satisfaction with labor and employment,” “satisfaction with social security,” “satisfaction with basic social services such as subsistence allowances and disasters,” “satisfaction with public culture and sports,” and “urban and rural infrastructure satisfaction.” To ensure the consistency of the variable values, this study converted the CGSS percentile answers: [90–100] = 5, [80–89] = 4, [70–79] = 3, [60–69] = 2, and [0–59] = 1.

#### Social Trust

According to the theory of social trust, trust refers to a general sense of confidence in the dependence of other people or systems in society. From this, the following variables were selected. In the social trust variable selection questionnaire, the options for “In general social interactions/contacts that do not directly involve monetary interests, do you think there are many people you can trust among your neighbors?” were “mostly untrustworthy” (= 1), “untrustworthy” (= 2), “half are trustworthy, and half are untrustworthy” (= 3), “trustworthy” (= 4), “mostly trustworthy” (= 5). For “Persons participating in recreational, fitness, training, and other leisure activities together,” the options were “mostly untrustworthy” (= 1), “untrustworthy” (= 2), “half are trustworthy, and half are untrustworthy” (= 3), “trustworthy” (= 4), and “mostly trustworthy” (= 5). For “People who participate in religious activities together,” the options were “mostly untrustworthy” (= 1), “untrustworthy” (= 2), “half are trustworthy, and half are untrustworthy” (= 3), “trustworthy” (= 4), and “mostly trustworthy” (= 5). For “Persons participating in social activities/public welfare activities together,” the options were “mostly untrustworthy” (= 1), “untrustworthy” (= 2), “half are trustworthy, and half are untrustworthy” (= 3), “trustworthy” (= 4), and “mostly trustworthy” (= 5). For “How familiar are you with your neighbors/neighborhood/other residents in the same village?” the options were “very unfamiliar” (= 1), “not very familiar” (= 2), “generally familiar” (= 3), “fairly familiar” (= 4), and “very familiar” (= 5).

### Reliability and Validity Test

Before the formal analysis of the sample data, the reliability and validity of the four scales of social class identity, public service satisfaction, social trust, and residents’ happiness were tested. The results are presented in Tables 1, 2. SPSS 23.0 was used to calculate Cronbach’s α coefficient of each variable.

**TABLE 1:** Reliability analysis of variables.

Variable	Number	Evaluation criterion	α Value	Total α value	Fitting
Social class identity	4	>0.60	0.801	0.828	Good reliability
Public service satisfaction	9	>0.60	0.939		
Social trust	5	>0.60	0.647		
Resident’s happiness	3	>0.60	0.601		

**TABLE 2:** Validity result analysis.

KMO value		0.895
Bartlett’s sphericity test	χ^2^ value	35721.372
	Degree of freedom	210
	*P-*value	0.000

From the results in Table 1, Cronbach’s α coefficient value of each variable is between 0.601 and 0.939 and exceeds 0.6, indicating that the sample data of the questionnaire has good reliability. The overall Cronbach’s α coefficient value is 0.828, which far exceeds 0.6 and indicates that the sample results have a certain degree of authenticity and provides a basis for further research.

Second, the KMO value of all variables were calculated. The results are presented in Table 2.

The data in Table 2 show that the KMO value is 0.895, which is much greater than 0.5, and the *p*- value of Bartlett’s sphere test is 0.000 < 0.05, indicating that the test effect is significant, and the validity of the scale is good.

### Descriptive Statistics and Correlation Test of Variables

Table 3 shows the results of descriptive statistics and correlation analysis of each variable. The empirical results show that there is a significant positive correlation between social class identification and residents’ happiness (*r* = 0.351, *p* < 0.01), hypothesis 1 has been initially verified. Social class identity and public service satisfaction show a significant positive correlation (*r* = 0.106, *p* < 0.01), hypothesis 2 has been initially verified. Social class identity and social trust are significantly positively correlated (*r* = 0.009, *p* < 0.05), hypothesis 3 has been initially verified. There is a significant positive correlation between public service satisfaction and social trust (*r* = 0.148, *p* < 0.01), hypothesis 4 has been initially verified. Social trust has a significant positive correlation with residents’ happiness (*r* = 0.072, *p* < 0.01), hypothesis 5 has been initially verified. Public service satisfaction has a significant positive correlation with residents’ happiness (*r* = 0.129, *p* < 0.01), hypothesis 6 has been initially verified (see Table 3).

**TABLE 3:** Descriptive statistics and correlation test of variables.

Variable	Social class identity	Public service satisfaction	Social trust	Resident’s happiness
Social class identity	1	0.106**	0.009*	0.351**
Public service satisfaction	0.106**	1	0.148**	0.129**
Social trust	0.009*	0.148**	1	0.072**
Resident’s happiness	0.351**	0.129**	0.072**	1
Mean	2.318	3.683	3.340	3.398
Standard deviation	0.698	0.746	0.661	0.611

*p < 0.05, **p < 0.01.

### Ethics Statement

This study was conducted in accordance with the Declaration of Helsinki, and the protocol was approved by the Ethics Committee (HREC) of the School of Economics and Management in East China Jiaotong University. The participants provided their written informed consent to participate in this study.

## Results

### Model Fit Degree Analysis

We used Amos22.0 to test the model. We conducted a model fit degree analysis (see Table 4). By referring to the fitting index standard defined by Wu and Ding (2004), the empirical results show that all measures have acceptable validity, Chi-square = 6158.94, DF = 1260, Chi-square/DF = 4.909 < 5.00, the adjusted goodness-of-fit index (AGFI) = 0.938 > 0.90, GFI = 0.952 > 0.90, root mean square error of approximation (RMSEA) = 0.051 < 0.08, comparative fit index (CFI) = 0.952 > 0.90, and incremental fit index (IFI) = 0.952 > 0.90. Therefore, the model-fitting level constructed in this study is acceptable.

**TABLE 4:** Model fitting index values.

Statistical tests	Fitting indicators	Evaluation criterion	Model results	
Absolute fitness index	GFI	>0.90	0.952	Ideal
	AGFI	>0.90	0.938	Ideal
	RMSEA	<0.08	0.051	Ideal
Value-added fitness index	NFI	>0.90	0.947	Ideal
	IFI	>0.90	0.952	Ideal
	CFI	>0.90	0.952	Ideal
Minimalist fitness index	PGFI	>0.50	0.741	Ideal
	PNFI	>0.50	0.812	Ideal
	PCFI	>0.50	0.816	Ideal

### Testing of Hypotheses

**FIGURE 2:**
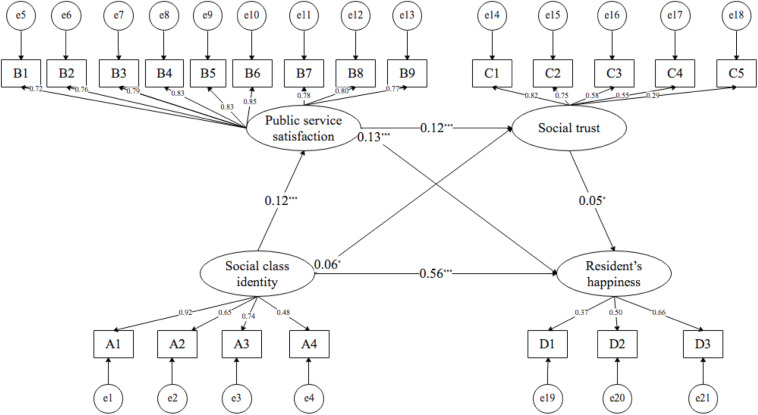
Structural equation modeling results. *P < 0.05, **P < 0.01, ***P < 0.001.

The hypotheses were tested by using structural equation modeling (see Figure 2).

First, social class identity can significantly improve residents’ happiness. The path coefficient of social class identity to residents’ happiness is 0.56 (*p* < 0.001). From the perspective of reference group theory, subjective happiness is often the result of social comparison. A higher social class identity often means higher income and non-income benefits, such as the establishment of prestige status, improvement of the quality of life, and access to social respect. All of these factors are conducive to enhancing residents’ happiness; thus, Hypothesis 1 is supported. Second, social class identity can significantly enhance public service satisfaction. The path coefficient of social class identity to public service satisfaction is 0.12 (*p* < 0.001). The improvement of social class identity often means that public service resources can be prioritized. Meanwhile, it is conducive to obtaining higher-quality public services, so social class identity has a direct positive effect on public service satisfaction. Thus, Hypothesis 2 is supported. Third, social class identification can significantly enhance social trust. The path coefficient of social class identification to social trust is 0.06 (*p* < 0.05). This shows that the higher an individual’s social class identity, the stronger the possible sense of control and happiness, the lower the risk perception of their own decision-making, the stronger the perception of future security, and the higher the level of social trust they hold. Thus, Hypothesis 3 is supported. Fourth, public service satisfaction has a positive impact on social trust. The path coefficient of social trust to public service satisfaction is 0.12 (*p* < 0.001). From the perspective of social exchange theory, there is an exchange relationship between individuals and the government. The starting point of the exchange is the individual’s expectations of the government’s ability and behavior. The individual provides resources, trust, and votes to the government in exchange for high-quality public services. Therefore, the higher a person’s satisfaction with public services, the higher their social trust in the government. Thus, Hypothesis 4 is supported. Fifth, social trust can significantly improve residents’ happiness. The path coefficient of social trust to residents’ happiness is 0.05 (*p* < 0.05). From the perspective of social capital, individuals who trust others are more likely to feel support from the outside world; that is, individuals with a higher level of social trust feel more social support, and individual’s happiness experiences may be higher. Thus, Hypothesis 5 is supported. Finally, public service satisfaction has a positive effect on residents’ happiness. The path coefficient of public service satisfaction to residents’ happiness is 0.13 (*p* < 0.001). Public service satisfaction has a direct positive effect on residents’ happiness. Thus, Hypothesis 7 is supported (see Tables 5).

**TABLE 5:** Hypothesis test results of path coefficients of structural equation model.

Variable	Standardized path coefficients	*P*	Hypothesis	Result
Social class identity — > residents’ happiness	0.560	0.000	1	Supported
Social class identity — > public service satisfaction	0.120	0.000	2	Supported
Social class identity — > social trust	0.06	0.003	3	Supported
Public service satisfaction — > social trust	0.12	0.000	4	Supported
Social trust — > residents’ happiness	0.05	0.032	5	Supported
Public service satisfaction — > residents’ happiness	0.120	0.000	6	Supported

We used the Monte Carlo analysis method with 10,000 resamples to test the mediation hypotheses (see Table 6). First, the results indicate that the modified confidence interval of Monte Carlo for the indirect effect of social class identity on residents’ happiness is (0.001, 0.003) at the 95% confidence level, zero is not within the range, and the *p*-value is less than 0.001, which indicates that the mediation effect of social trust between social class identity and residents’ happiness is significant. Moreover, the Monte Carlo confidence interval of the direct effect of social class identity on residents’ happiness is (0.270, 0.343), zero is not within the range, and the *p*-value is less than 0.001, which further indicates that social trust plays a partial mediation effect between social class identity and residents’ happiness. Thus, Hypothesis 6 is supported. Second, the results indicate that the modified confidence intervals of Monte Carlo for the indirect effect of social class identity on residents’ happiness is (0.004, 0.011) at the 95% confidence level, zero is not within the range, and the *p*-value is less than 0.001, which indicates that the mediation effect of public service satisfaction between social class identity and residents’ happiness is significant. Moreover, the Monte Carlo confidence interval of the direct effect of social class identity on residents’ happiness is (0.270, 0.343), zero is not within the range, and the *p*-value is less than 0.001, which further indicates that public service satisfaction plays a partial mediation effect between social class identity and residents’ happiness. Thus, Hypothesis 8 is supported.

**TABLE 6:** Monte Carlo mediation effects testing.

Summary of the hypothesized path	Coefficient	Monte Carlo 95% CI		*P*
		LL	UL	
**Gross effect**				
Social class identity — > residents’ happiness	0.314***	0.278	0.352	0.000
**Direct effect**				
Social class identity — > residents’ happiness	0.305***	0.270	0.343	0.000
**Indirect effect**				
Social class identity — > social trust— > residents’ happiness	0.001***	0.001	0.003	0.000
Social class identity — > public service satisfaction — > residents’ happiness	0.007***	0.004	0.011	0.000

CI, confidence interval; LL, lower limit; UL, upper limit. ***p < 0.001.

### Comparison of Differences Between Multi-Group Models

The fitting of the above model was based on the assumption of homogeneity for all groups. However, the internal structures of all the groups were heterogeneous. Therefore, it was necessary to compare the differences between the groups of different birth years and different types of household registration to reveal the differences between different groups more intuitively and accurately. This study divided the sample into those born in the 1960s (1960–1969), the 1970s (1970–1979), the 1980s (1980–1989), and the 1990s (1990–1999) as well as agricultural households, according to the year of birth and the type of household registration. Birth years were grouped at 10-year intervals, which can better distinguish different years and make the research results more scientific and reasonable.

Table 6 reflects the degree of fit between the different group models and the surveyed data (see Table 7). The empirical results show that the GFI indexes of the two models of those born in the1960s and the 1990s populations meet the evaluation criteria, showing that the fitting indexes of the two models are ideal. However, in the two models for those born in the 1970s and the 1980s groups, the AGFI value is slightly lower than the evaluation standard, showing that the fitting of the two models is good. The parameter values of the two models of agricultural household registration and non-agricultural household registration are within the reference range, showing that the two models fit perfectly. Therefore, the fitting levels of the five different group models constructed in this study are acceptable.

**TABLE 7:** Comparison of parameters between different groups.

Variable	Different population	GFI	AGFI	RMSEA	TLI	Result
		**>0.90**	**>0.90**	**<0.08**	**>0.90**	
Different age groups	1960s	0.941	0.925	0.020	0.930	Ideal
	1970s	0.921	0.893	0.077	0.917	Preferable
	1980s	0.922	0.896	0.076	0.910	preferable
	1990s	0.934	0.911	0.066	0.933	Ideal
Type of account	Agricultural	0.944	0.924	0.067	0.933	Ideal
	Non-agricultural	0.941	0.921	0.068	0.937	Ideal

*“Ideal” means that the fitting index is within the reference value range; “good” means that the fitting index is not within the reference value range but is slightly lower or slightly higher.*

From the different age groups, we compared the effects and path coefficients between social class identities, social trust, public service satisfaction, and residents’ happiness, as shown in Table 8. The coefficients in the Table 8 are standardized.

**TABLE 8:** Comparison of path standardization coefficient between different groups.

Path	Different age groups				Type of account	
	1960s	1970s	1980s	1990s	Agricultural	Non-agricultural
Social class identity — > residents’ happiness	0.51***	0.46***	0.25***	0.17**	0.31***	0.56***
Social class identity — > public service satisfaction	0.26***	0.25***	0.13*	0.15*	0.18***	0.26***
Social class identity — > social trust	0.06	0.12	0.10	0.01	0.14***	0.08
Public service satisfaction — > social trust	0.16***	0.10*	0.09	0.12	0.11***	0.16***
Social trust — > residents’ happiness	0.01	0.13	0.08**	0.02	0.01	0.07***
Public service satisfaction — > residents’ happiness	0.08	0.08**	0.09***	0.05	0.06***	0.09***

*p < 0.05, **p < 0.01, ***p < 0.001.

First, the influence coefficient of social class identity on residents’ happiness in those born in the 1960s and the 1970s groups are greater than those born in the 1980s and the 1990s groups. Those born in the 1990s group’s social class identity has the smallest impact coefficient on residents’ happiness, which is only 0.17. A possible reason is that those born in the 1990s group are relatively young and most are in the career struggle period, so they have not yet achieved a higher social class. Those born in the 1970s group are aged between and 40–50 years old, and some are almost 50 years old. According to Maslow’s hierarchy of needs theory, the first three levels of physiological needs, safety needs, and emotional and belonging needs are often met (Koltko-Rivera, 2006). This group generally hopes to further realize a higher social class and meet the fourth-level needs of respect and the fifth-level needs of self-realization. Therefore, the increase in social class identity in those born in the 1970s group significantly contributes to the improvement of residents’ happiness. Second, the influence of social class identity in those born in the 1960s and the 1970s groups on public service satisfaction is greater than that of those born in the 1980s and the1990s groups. A possible reason is because those born in the 1990s are younger, and most of them are in the career struggle period and have not achieved a higher social class. Those born in the 1960s and the1970s groups already have a stable and high social class, meaning access to high-quality public services and happiness. Therefore, social class identity has a greater impact on public service satisfaction.

Third, the social class identity of the non-agricultural household registration has a greater impact on happiness than the agricultural household registration, the social class identity of the non-agricultural household registration is more satisfied with public services than the agricultural household registration, and the public service satisfaction of the non-agricultural household registration has a greater impact on happiness than the agricultural household registration. A possible reason is because for non-agricultural *hukou* (household registration system) residents, in urban areas where talents gather, they need to compete fiercely to gain a higher social class identity. This can often bring more direct and effective happiness to residents.

## Discussion

This study uses the data of CGSS to examine the influence of social class identity and public service satisfaction on residents’ happiness. We analyzed the descriptive statistical characteristics of the sample; established a structural equation model to explore the impact of social class identity and public service satisfaction on residents’ happiness; and adopted the Monte Carlo method to test the mediating effect of public service satisfaction and social trust between social class identity and residents’ happiness. The results showed that social class identity, social trust, and public service satisfaction have a positive impact on residents’ happiness, and the influence of social class identity on residents’ happiness is higher than that of public service satisfaction on residents’ happiness. Public service satisfaction and social trust have significant partial mediation effects on social class identity and residents’ happiness. These conclusions are basically consistent with previous studies (Yin et al., 2019; Xu et al., 2020). In addition, part of the total effect of social class identity on residents’ happiness comes from the direct effect of social class identity on residents’ happiness; another part comes from the indirect effect of public service satisfaction and social trust on residents’ happiness. There are significant differences in residents’ happiness between the different attribute groups. In those born in the 1960s and the 1970s groups, the influence coefficient of social class identity on residents’ happiness and on public service satisfaction is greater than that of those born in the 1980s and the 1990s groups. The social class identity of non-agricultural household registration has a greater impact on happiness and public service satisfaction than the agricultural household registration.

### Implication for Research and Practice

The study makes the following theoretical contributions:

First, from the perspective of social resource theory, reference group theory, and happiness economics theory, this study explains the positive influence mechanism of social class identity, public service satisfaction, and social trust on residents’ happiness. In recent years, many scholars have begun to pay attention to the impact of social class identification and public service satisfaction on residents’ happiness, but they are mostly limited to the single study of social class and residents’ happiness or public service satisfaction and residents’ happiness. There is a lack of research on the integration of the three, and the internal mechanism has not been clarified. This study reveals not only that social class identity has a positive impact on residents’ happiness, but also that there exists the mediating role of public service satisfaction and social trust between social class identification and residents’ happiness.

Second, while domestic and foreign scholars have carried out related research on the process mechanism of social class identity affecting residents’ happiness (Yamamura et al., 2014; Dodell-Feder et al., 2019), this study found that public service satisfaction plays a significant intermediary role in the process of social class identity affecting residents’ happiness. This research not only incorporates public service satisfaction into the research framework of the impact of social class identity on residents’ happiness, but also introduces social trust variables to jointly explore the internal mechanism of social class identity affecting residents’ happiness. According to social exchange theory, there is an exchange relationship between people and the government. The starting point of the exchange is the people’s expectations of the government’s capabilities and behaviors. People provide the government with resources, trust, votes, and so on in exchange for the government’s quality public services (Tao et al., 2011). Higher the people’s satisfaction with the public services provided by the government, higher their trust in the government. To sum up the current study, it expands the research context of the factors influencing residents’ happiness and enriches and deepens research results related to residents’ happiness.

Third, the study refines both the different birth years into the 1960s, 1970s, 1980s, and 1990s groups and the different types of household registration into agricultural and non-agricultural to identify the different effects of social class identity, social trust, and public service satisfaction on residents’ happiness according to different birth years and different types of household registration. Specifically, the influence coefficient of social class identity on the happiness of those born in the 1960s and the 1970s groups is greater than that of those born in the 1980s and the 1990s groups. Those born in the 1990s group is young, with most participants in the career struggle period, and most of them have not yet achieved a higher social class. For those participants born in the 1970s group, which is between 40–50 years old, their three levels of physiological needs, safety needs, and love and belonging needs have often been met, according to Maslow’s hierarchy of needs theory (Koltko-Rivera, 2006). This group generally hopes to further achieve a higher social class and meet the fourth level of esteem needs.

Moreover, the study provides the following practical implications based on the findings:

First, to provide a basic public service system that allocates public resources in a fair and reasonable way, government administrators should solve livelihood issues, such as employment, social security, public education, and medical care, and improve residents’ satisfaction with public services to improve their happiness.

Second, social managers should create an equal and fair institutional environment, make new breakthroughs in the equalization of public services, guide people to improve their awareness of happiness, and improve the mental health of residents.

Third, the government should further transform from the economic construction type to the public service-oriented type, promote urban-rural integration, and make efforts to improve both the public service level and the happiness of rural residents.

Fourth, social trust is of great significance in improving residents’ happiness. Rebuilding social trust is an inevitable requirement for building a harmonious society and ensuring and improving people’s livelihoods. On one hand, the government must improve laws and regulations and regulate its own behavior and market order; on the other hand, it must strengthen the construction of trust ethics, guide the shaping of social trust habits, and build a trust culture. Only in this way can people restore their confidence in the government, market, and society, and effectively enhance their happiness.

### Limitations

First, we collected data from the CGSS, which has limitations concerning the provinces covered by the sample. In future, we can combine China Family Panel Studies (CFPS) data with CGSS data for further analysis.

Second, although we discuss differences in social class identity, social trust, and public service satisfaction affecting residents’ happiness using different birth years and different household registration groups, we failed to consider how different genders or different marriage groups may have affected the differences. Thus, we recommend that future researchers obtain a more extensive dataset and investigate how different genders or different marriage groups may affect differences in social class identity, public service satisfaction, and social trust affecting residents’ happiness.

## Data Availability Statement

The original contributions presented in the study are included in the article/supplementary material, further inquiries can be directed to the corresponding author/s.

## Ethics Statement

The studies involving human participants were reviewed and approved by the Ethics Committee of School of Economics and Management in East China Jiaotong University. Written informed consent for participation was not required for this study in accordance with the national legislation and the institutional requirements.

## Author Contributions

XZ and SC designed the research and methodology, compiled the literature, and put forward the policy recommendations. XZ provided guidance throughout the entire research process. LC and LL revised and approved the manuscript. All authors contributed to the article and approved the submitted version.

## Conflict of Interest

The authors declare that the research was conducted in the absence of any commercial or financial relationships that could be construed as a potential conflict of interest.
